# DNA Repair and the Stability of the Plant Mitochondrial Genome

**DOI:** 10.3390/ijms21010328

**Published:** 2020-01-03

**Authors:** Nicolas Chevigny, Déborah Schatz-Daas, Frédérique Lotfi, José Manuel Gualberto

**Affiliations:** Institut de biologie moléculaire des plantes, CNRS, Université de Strasbourg, 67081 Strasbourg, France; nicolas.chevigny@etu.unistra.fr (N.C.); deborah.schatz2@etu.unistra.fr (D.S.-D.); frederique.lotfi@ibmp-cnrs.unistra.fr (F.L.)

**Keywords:** mitochondria, mtDNA, homologous recombination, base excision repair

## Abstract

The mitochondrion stands at the center of cell energy metabolism. It contains its own genome, the mtDNA, that is a relic of its prokaryotic symbiotic ancestor. In plants, the mitochondrial genetic information influences important agronomic traits including fertility, plant vigor, chloroplast function, and cross-compatibility. Plant mtDNA has remarkable characteristics: It is much larger than the mtDNA of other eukaryotes and evolves very rapidly in structure. This is because of recombination activities that generate alternative mtDNA configurations, an important reservoir of genetic diversity that promotes rapid mtDNA evolution. On the other hand, the high incidence of ectopic recombination leads to mtDNA instability and the expression of gene chimeras, with potential deleterious effects. In contrast to the structural plasticity of the genome, in most plant species the mtDNA coding sequences evolve very slowly, even if the organization of the genome is highly variable. Repair mechanisms are probably responsible for such low mutation rates, in particular repair by homologous recombination. Herein we review some of the characteristics of plant organellar genomes and of the repair pathways found in plant mitochondria. We further discuss how homologous recombination is involved in the evolution of the plant mtDNA.

Excellent reviews on how recombination mechanisms shape the structure of plant mitochondrial genomes have been published, several of them in the last few years [1,2,3,4]. Recently, we have also addressed possible recombination pathways and mutations that can explain the rapid evolution of plant mitochondrial genome structures [5]. In this present review that is to be part of a special issue of the *IJMS* on “*DNA Damage and Repair in Plants*” we revisit the subject, but with a special emphasis on the repair pathways that exist in plant mitochondria.

## 1. Origin, Size, and Coding Capacity of the Organellar Genomes

The mitochondrial and chloroplast genomes (mtDNA and cpDNA) are derived from the genomes of the symbiotic ancestors of the two organelles: an α-proteobacteria and a cyanobacteria, respectively [6,7,8]. During their evolution the organellar genomes were gradually reduced via gene loss or migration to the nuclear genome. At present, the mtDNA is highly variable in size and structure depending on the species, and codes for just a few genes. Thus, the human mtDNA is a 16.6-kb circular molecule that contains 37 genes, encoding 13 proteins involved in the electron transport chain, 22 tRNAs, and two rRNAs. That corresponds to a 98.5% reduction in size and 95% reduction in coding capacity as compared to the genome of *Rickettsiale* bacteria, obligatory parasites that apparently are close to the proto-symbiont ancestor of mitochondria [9]. In yeast, the mtDNA can be as small as 18.5 kb in *Hanseniaspora uvarum* and can reach more than 105 kb in *Kluyveromyces bacillisporus*, with a coding capacity similar to the mtDNA of animals (30 to 40 genes) [10,11,12]. Simpler organisms can have a higher number of mitochondrial genes, with the notable example of the protist *Reclinomonas americana* that has a 69-kb mtDNA coding for about 100 genes [13]. Other protists, such as *Trypanosoma brucei* or *Diplonema papillatum*, can have huge mtDNA genomes (4.2 Mb and 260 Mb, respectively), without encoding more proteins than the mtDNA of animals [14]. In land plants, the mtDNA is large in size, typically around 200–400 kb, but it can be much bigger, reaching up to 11.3 Mb in the case of *Silene conica* [15]. In spite of their size the plant mtDNA only has about 20 additional genes in comparison to animals and yeasts. Thus, the 367 kb mtDNA of Arabidopsis only encodes 32 proteins (involved in the electron transport chain and the mitoribosome structure), 22 tRNAs, and three rRNAs (5S, 18S, and 26S) [16]. The vast majority of the mtDNA is composed of non-coding sequences derived from nuclear (4%), chloroplast (1%), viral, or unknown sequences for the most part (62%). These proportions are much larger for very large genomes such as those of *Silene*, which encode even fewer genes.

Although the plant mitochondrial genomes vary in size and gene content among species, in most seed plant species the gene-coding sequences evolve very slowly, with synonymous substitutions rates 100 lower than in animal mitochondria [17]. As compared to the nuclear and chloroplast genomes, plant mitochondrial substitution rates are 10 and 3–4 lower, respectively [18,19,20]. However, there are notorious exceptions, as in *Geraniaceae*, *Plantaginaceae*, *Silene*, and *Ajuga* [21,22,23,24,25,26]. In contrast to the high variability in size and gene content of the mtDNA, the cpDNA of higher plants is quite conserved in size and coding capacity, typically 150 kb in size for 120 genes (versus 4 Mb and 3000 genes for the genomes of cyanobacteria) [27].

## 2. Structure of Plant Organellar Genomes

The mitochondrial as well as the chloroplast genomes are packaged in nucleoprotein complexes called nucleoids, similar to those of bacterial chromosomes [28,29]. These nucleoids are compared to protonuclear structures because, like the nucleus, they are the seat of replication and transcription [30]. They are also proposed to be the sites of rRNA processing and ribosome assembly [31]. In mitochondria, nucleoids are located in the matrix and can be exchanged between mitochondria during fusion and fission events [32]. In a typical human cell there are 1000 and 5000 copies of the mtDNA, and super-resolution microscopy revealed that there are only one or two copies of the mtDNA per nucleoid [33], which is structured by the DNA-binding protein mitochondrial transcription factor A (TFAM) [34]. In plants, the exact composition of organellar nucleoids is not yet fully resolved, but many of the factors involved in the expression, maintenance, and segregation of the organelle genomes have been associated with these complexes [35,36,37]. Chloroplast nucleoids from several species have been purified and their composition is better known [35,37,38]. They are composed of many factors involved in the replication, transcription, organization, and repair of cpDNA, but also in the processing of transcripts (splicing and editing) and ribosome biogenesis. The presence of these factors within the nucleoids could indicate a co-transcriptional processing of transcripts coupled directly to translation, in the same way as in prokaryotes [35,37]. Anchor factors for nucleoids with chloroplast membranes have also been found [30,39]. A mature chloroplast has about 20 nucleoids, with an average ploidy of 4–5 plastid genomes per nucleoid [40]. Although the plant mitochondrial nucleoid is less described, among other components it probably contains the organellar PolI-like DNA polymerases POL1A and POL1B, the replicative DNA primase-helicase TWINKLE, the type II topoisomerase Gyrase, the RecA-like recombinases RECA2 and RECA3, the SSB-like ssDNA-binding proteins SSB1 and SSB2, several other DNA binding proteins, the phage-type RNA polymerases RPOTm and RPOTmp, and the MutS-like homolog MSH1 [41,42]. Many of these proteins are also involved in DNA repair and homologous recombination.

The mtDNA of higher plants is rich in repeated sequences. These can be involved in homologous recombination events and consequently have a major impact in the structure of the mtDNA, as specifically discussed in other reviews [2,4,5]. These mtDNA repeats can be classified into large repeated sequences (several kilobases), intermediate-size repeated regions (50 to 500 bp), and microhomologies (<50 bp). Large repeats are often involved in reversible reciprocal recombination events that modulate the plasticity of the plant mtDNA, typically composed of a collection of subgenomes interconvertible by recombination. Two pairs of large repeats are present in the mtDNA of Arabidopsis accession Col-0, but many large repeats can be present in the mtDNA of other species. The plasticity of the mtDNA by recombination makes it difficult to model the structure of the mtDNA. It is usually possible to map all the mtDNA sequences in a single circular chromosome called the master circle, but such genome-size circular molecules have only been observed in very few species, in relatively old reports [43,44], and such observations were not reproduced [45]. Rather, pulse field gel electrophoresis and microscopy studies have shown that the plant mtDNA is apparently composed of a mixture of circular subgenomes and linear and branched molecules. Work from Backert and collaborators showed that most large mtDNA molecules observed by electron microscopy are linear or rosette-like, while most circular molecules are of small size [46,47]. An important proportion of the genome was also found to be single-stranded [48]. Structural analysis using moving pictures and pulsed field gel electrophoresis (PFGE) also suggested that the plant mtDNA mainly exists as complex branched structures or simple linear molecules derived from the complex forms [49]. Recent analysis of the mtDNA from lettuce also showed similar mtDNA structures [50]. An extensive and historical review on the studies concerning the size and structure of the plant organellar genomes is presented elsewhere [51]. It is therefore likely that the genome is a collection of subgenomes produced by recombination between direct repeats within one or several master chromosomes. It is also possible that the genome is mainly constituted by circularly permuted linear molecules containing overlapping ends, allowing mapping of the genome into a circle, even if such structure does not exist in vivo [52]. Some of the linear molecules might be also concatamers of the genome. The existence of concatemeric mtDNA is supported by the presence of an insertion derived from a mitochondrial genome concatamer in the Arabidopsis nuclear genome [53].

In contrast to the recombination involving large repeats, the ectopic recombination between intermediate-size repeats and microhomologies is infrequent, usually asymmetric, and not reversible [1,54,55]. These rare recombination events contribute to the heteroplasmic state of the mtDNA, and are responsible for the different populations of mtDNA, called sublimons, that can co-exist in the same individual, often less than one copy per cell [54,56]. Consequently the plant mtDNA sequences are not uniformly conserved, and some parts can be stoichiometrically more represented than others in a cell [57]. One cell also has more mitochondria than mtDNA copies, and qPCR quantification indicated that some mitochondria do not contain any DNA [57]. In agreement, it was also observed that during mitochondria division not all daughter mitochondria inherit nucleoids, and that DNA-containing mitochondria not necessarily contain a complete set of the genome [32,58]. Taken together, the available data suggest that plant mtDNA is divided into several subgenomic molecules obtained by recombination events, dispersed in the different mitochondria of a cell according to their fusion and fission.

But how such a collection of linear or circular subgenomes replicates is still not understood. It was proposed that the plant mtDNA replicates by mechanisms of recombination-dependent replication similar to those of certain bacteriophages [59,60]. However, the study of mitochondrial plasmids that can exist in the mitochondria of certain plant species also revealed processes of rolling circle replication apparently initiated at R-loops [61]. An additional argument for circular replicating subgenomes is the recent observation that specific cutting of mtDNA sequences with transcription activator-like effector nucleases (TALEN) results in the deletion of the targeted sequences and surrounding regions [62], as expected if the targeted sequences are contained in subgenomes that no longer replicate if a double-strand break is introduced. Mutants of recombination factors also revealed the autonomous replication of circular subgenomes generated by recombination, although the underlying replication mechanisms remained elusive [63]. The set of mitochondrial proteins already identified as potentially involved in mtDNA replication supports the idea that replication might follow several processes, including recombination-dependent replication mechanisms, but also replication by a replisome similar to the one of bacteriophages [3,64,65]. Such a replisome would require primers, which could be synthesized by the TWINKLE replicative helicase that also has primase activity [66,67].

Regulation of mtDNA copy numbers might be also regulated according to the type of cell and developmental state. In Arabidopsis it varied between organs and changed during the development of cotyledons and leaves [57]. In rice an order of magnitude higher mtDNA copy number was found in egg cells as compared to leaf protoplasts [68]. However, in Arabidopsis and tobacco few mtDNA copy numbers were determined in egg cells, equivalent to those found in mesophyll cells [69]. In the same study a very large mtDNA content was found in egg cells of *Pelargonium zonale*, implying that the mtDNA can be selectively amplified in the generative cells of some species [69]. In contrast, a dramatic decrease was observed during pollen development, with an estimated 50-fold reduction in the generative cell, less than one mtDNA copy per cell [69,70]. Dismissal of the mtDNA in germinative pollen would be because of selective degradation, and several nucleases have been identified that could be involved [71]. The degradation of organellar DNA by exonucleases could be also a mean for rapid access to phosphate when plants are exposed to nutrient-deficient conditions [72].

## 3. Repair Mechanisms in the Mitochondria of Plants

Initially, it was thought that organelles did not have mechanisms for DNA repair, and that the high ploidy of the mtDNA prevented that damage to one copy would not be deleterious to the cell [73]. The damaged copies would rather be destroyed and other mtDNA copies produced by replication. However, it is now well established that DNA repair does exist in organelles, although not all pathways, and is not the same in different species [74,75]. Thus, the mitochondrial genomes of animals show a higher mutation rate than their nuclear genomes, leading to their rapid evolution [76]. This high mutation rate is partially due to the highly oxidative environment in the mitochondrial matrix that damages the mtDNA, and to the low fidelity of the animal mitochondrial DNA polymerase. The few DNA repair mechanisms that exist in animal mitochondria are probably an additional reason for the high mutation rate of the animal mtDNA. In contrast, in most higher plant species the mtDNA sequences are highly conserved and evolve very slowly, even if the organization of the genome is highly variable [17,18]. The importance of mitochondrial repair mechanisms has been put forward to explain such lower mutation rates, in particular repair by homologous recombination [77,78].

Herein, we review some of the repair pathways that exist in plant mitochondria. Some of these repair mechanisms and enzymes are common to those found in the nucleus [79]. We focus on those better studied that have unequivocally been demonstrated to be present in plant mitochondria, which are base excision repair (BER) and homologous recombination (HR).

### 3.1. Direct Repair

Typical UV-induced lesions are formed by the bridging of two pyrimidines within the same DNA strand, which causes a significant deformation of the DNA helix [80]. This deformation blocks the progression of enzymes moving along the DNA, and can cause double-strand breaks (DSBs) during the collision of the replication machinery with a blocked transcription elongation complex. The direct reversal of damage, or photo-reactivation, restores the structure of the DNA helix without excising the modified nucleotides. It relies on the use of blue light by photolyases that by electron transfer revert the bridging between the two pyrimidines [81]. UV radiation also induces the formation of pyrimidine dimers in organelle genomes [82].

The existence of direct repair was shown, in the mitochondria of *Saccharomyces cerevisiae* and in the chloroplast of *Chlamydomonas reinhardtii* [83,84]. However, its presence in the organelles of higher plants is controversial, and several studies on different plant species failed to detect photo-reactivation activity associated to organelles [82,85]. Yet, Takahashi et al. (2011) showed that in rice a nuclear photolyase is also directed to mitochondria and chloroplast, and a photolyase was also identified in Arabidopsis organelles [86,87]. However, the DNA repair activity of these proteins has not been shown, and they might have other functions as photoreceptors.

### 3.2. Mismatch Repair (MMR)

MMR allows the recognition of incorrect or modified nucleotides incorporated into the DNA during replication. In prokaryotes, it is the MutS protein that specifically recognizes and binds to the mutated base on the newly formed DNA strand. The MutL protein then stabilizes the MutS-DNA complex to allow the action of the MutH endonuclease, which incises the DNA strand upstream or downstream of the lesion. Exonucleases are then recruited to digest the DNA till the mismatch and DNA polymerase fills the DNA gap [88,89]. In plants, a MutS homolog (MSH1) is targeted to mitochondria and chloroplasts, but no other MMR factors has been identified in organelles. However, MSH1 has a C-terminal GIY-YIG homing endonuclease domain, which could allow it to incise DNA, as MutH [90]. This hypothesis still needs to be confirmed: the GIY-YIG domain of MSH1 was found to bind to branched DNA structures, but exhibited no endonuclease activity by itself in vitro [91]. However, it is possible that a nuclease activity can only be restored in the context of the whole protein or in interaction with additional factors.

The absence of MSH1 results in rearrangements of mtDNA and cpDNA, suggesting a role in recombination processes [42,92]. It was suggested that MSH1 destabilizes recombination intermediates when it detects loss of sequence homology [42,90]. According to Christensen, MSH1 could promote the repair of mismatches by inducing double-strand break (DSB) at the mutated base [2,5]. The organelle recombination machinery would then repair these DSBs, thus allowing the copy-correction of the lesion. This process would also account for the low mutation rate of the organelle genomes. This model is in agreement with the known importance of MMR observed in other non-organellar genetic systems, in post-replication repair and in the policing of homologous recombination [88].

### 3.3. Nucleotide Excision Repair (NER)

Lesions deforming the DNA helix can also be repaired by the NER pathway, which recognizes and removes DNA-binding lesions and adducts that prevent the flow of the factors that need to move along the DNA [93]. NER can be divided into two pathways: global genomic NER and transcription-coupled NER, which preferentially repairs transcribed regions [94]. Only the damage recognition step differs between the two pathways, which then use the same factors. In prokaryotes global genomic NER relies on UvrA2-UvrB2 hetero-tetramers that randomly scan the genome continuously for DNA-helix-distorting lesions and on the endonuclease UvrC that cleaves the DNA on both sides of the damage. Transcription-coupled NER additionally requires transcription repair coupling factor (TRCF), a translocase that specifically recognizes RNA polymerases stalled by DNA lesions [95].

The NER pathway seems to be absent in organelles. No homologs of UvrA, UvrB, or UvrC are encoded by the genomes of flowering plants, nor are any factors involved in nuclear NER predicted as directed to the mitochondria or chloroplast. Surprisingly, a homolog of bacterial TRCF is coded by the genome of Arabidopsis, predicted to be targeted to the chloroplast [5]. However, in the absence of additional NER factors it might have evolved to assume other functions. For instance, it could be involved in the BER pathway, as has been proposed in animals for nuclear NER factors found to be addressed to mitochondria [96,97,98].

### 3.4. Base Excision Repair (BER)

**Figure 1:**
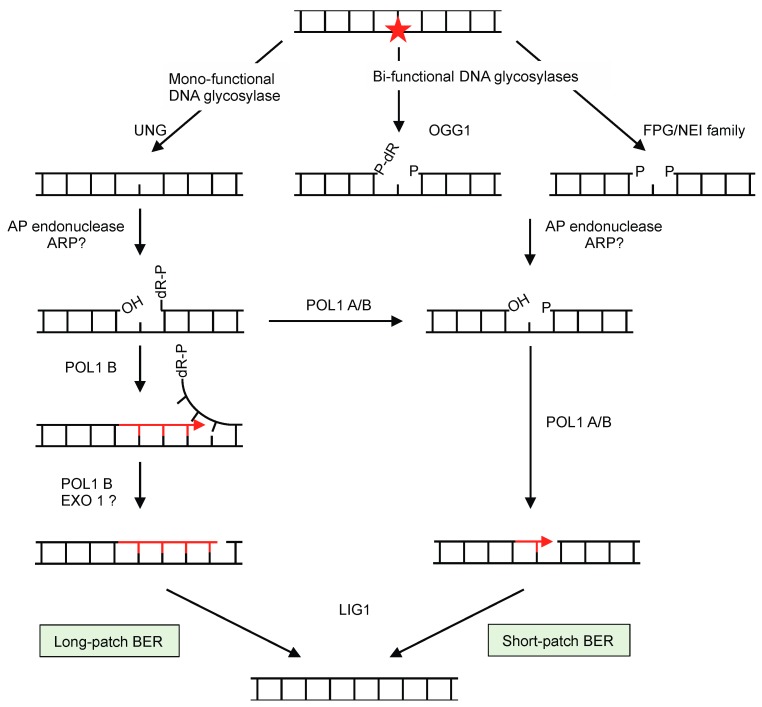
Mechanisms of base excision repair (BER). BER involves recognition and removal of base lesions (star) by glycosylases, and depending on the lesion and type of glycosylase it either follows the long-patch or short-patch pathways. Monofunctional glycosylases generate apurinic/apyrimidinic (AP) sites, while bifunctional glycosylases have AP lyase activity and leave a nucleotide gap with either a 3′ blocking sugar (P-dR, after 8-oxoguanine glycosylase OGG1 activity) or with 5′- and 3′-phosphate (P) groups at the termini (after the action of glycosylases of the formamidopyrimidine glycosylase/endonuclease VIII (FPG)/NEI) family). The activity of AP-endonuclease on the gaps left by bifunctional glycosylases will leave them ready for filling in and ligation by DNA polymerase (DNAP) and ligase, respectively, by short-patch repair. The AP sites left by monofunctional glycosylases are cleaved at the 5′- side by AP endonuclease, which leaves 3′-OH and 5′-P termini. Strand displacement by DNAP replaces 2–10 nucleotides by the long-patch pathway, and a nuclease is required to cleave the 5′dR-P (EXO1). Alternatively, long-patch intermediates can be converted to short-patch ones by the activity of the DNAP.

Oxidized, deaminated, and alkylated bases in the DNA are taken in charge by the BER pathway [99]. It recognizes and repairs lesions that moderately deform the double helix. The different steps of the alternative BER pathways are shown in Figure 1. It is well established that BER pathways exist in animal mitochondria, and there is strong evidence that plant mitochondria have them too [75].

The BER is initiated by the glycolytic excision of the damaged base, and its efficiency is based on the diversity of DNA glycosylases that specifically recognize different types of lesions [100]. Glycosylases targeting non-oxidative damages are mono-functional, while others that possess an associated lyase activity are classified as bi-functional [101] (Figure 1). A typical mono-functional DNA glycosylase is uracil-DNA glycosylase (UNG), which recognizes uracil in DNA resulting from misincorporation of dUMP during replication or from cytosine deamination. There is compelling evidence that UNG is present in the mitochondria of plants and contributes to the repair of the mtDNA. In vivo targeting of UNG-GFP fusions and in vitro import assays showed that UNG from Arabidopsis is imported into mitochondria [102]. UNG activity was also found associated to maize and potato mitochondrial fractions [103,104], and in organello experiments revealed UNG in the mitochondria of Arabidopsis, potato, and in the gymnosperm *Araucaria angustifolia* [102,105].

The bi-functional DNA glycosylases 8-oxoguanine glycosylase (OGG1) and the members of the formamidopyrimidine glycosylase/endonuclease VIII (FPG/NEI) family are able to take in charge the major oxidative lesions. It was shown that the simultaneous deficiency of FPG and OGG1 enzymes in Arabidopsis increases oxidative DNA damage in both the nuclear and mitochondrial genomes to a similar extent, strongly suggesting that BER dependent on these glycosylases exists in mitochondria [106]. OGG1 recognizes oxidative products of guanine, in particular 8-oxoguanine (8-OxoG), and catalyzes their excision. OGG1 from different plant species are predicted to be targeted into mitochondria, such as those of *Medicago truncatula*, *Populus nigra*, *Oriza sativa,* and Arabidopsis [107]. However, Arabidopsis OGG1 fused to YFP was found in the nucleus [108]. Nevertheless, the nuclear localization of most cellular OGG1 does not exclude the fact that some might be targeted to organelles. Accordingly, Ferrando et al. (2019) recently showed OGG1 activity associated to pure mitochondria isolated from potato tubers [104]. To overcome mtDNA oxidative damage and enhance mitochondrial activity this enzyme might perhaps be relocated to plant mitochondria containing elevated mtDNA oxidation, as occurs in *Saccharomyces cerevisiae* for *N*-glycosylase1 Ntg1 [109].

The FPG/NEI family members recognize oxidized pyrimidines, as well as formamidopyrimidine (FaPy), and oxidation products of 8-oxoG such as spiroiminodihydantoin and guanidinohydantoin. The substrate specificity and the cell localization of the different FPG isoforms still need to be analyzed, and it is therefore not clear if FPG/NEI exist in plant organelles. The *Medicago truncatula* and Arabidopsis FPG possess a nuclear localization signal [107,110], but the *FPG* gene can be expressed into seven different mRNAs by alternative splicing [111] and it is possible that some code for organellar isoforms of FPG. The presence of NEI glycosylase activity has recently been characterized in mitochondrial extracts of potato and *A. angustifolia* [104,105]. Interestingly, Cordoba-Canero et al. (2014) found an increase in oxidative damage in both nuclear and mitochondrial DNA from *fpg ogg1* Arabidopsis double mutants, but not in single mutants, suggesting that one glycosylase can compensate the absence of the other [106]. That is however surprising, because FPG is not supposed to recognize and excise 8-OxoG, the major substrate of OGG1 [112,113].

The abasic sites left by glycosylases are cleaved by an AP endonuclease, leading to a 5′-deoxyribose phosphate (5′-dR-P) moiety in the case of the products of monofunctional glycosylase, or to 5′-OH and 3′-P termini after action of a bifunctional glycosylase (Figure 1). AP-endonuclease activity was detected in Arabidopsis, potato, and *A. angustifolia* mitochondria [102,104,105], and the enzyme might be the product of the apurinic endonuclease-redox protein (*ARP*) gene. Interestingly, Ferrando et al. (2019) detected an induction of the potato mitochondrial APE1-like activity under hypoxia [104].

DNA lesions processed by bifunctional glycosylases are repaired following the short-patch BER pathway (single nucleotide replacement), while those recognized by monofunctional glycosylases can alternatively be repaired following long-patch (up to 10 nucleotide replacements) or short-patch BER [99]. In either case the activity of a DNA polymerase is required. Genes encoding plant organellar DNA polymerases are phylogenetically related to *Escherichia coli* DNA polymerase I [114,115]. In Arabidopsis and tobacco there are two, POL1A and POL1B, which are both dually targeted to mitochondria and chloroplasts [116,117,118,119]. These plant DNA polymerases can replicate an entire organellar genome and show high processivity without accessory proteins [64,120]. They have a fidelity ranking between those of replicative and lesion bypass DNA polymerases [121]. Initial characterization suggested that POL1A is only involved in replication and that POL1B has an additional role in DNA repair [36,122]. Recently it was shown that both enzymes present a 5′-dR-P lyase activity that can remove the 5′-dR-P moiety formed during short patch repair [123] (Figure 1). It was also shown that POL1B performs efficient strand-displacement on DNA containing a one-nucleotide gap, whereas POL1A has only moderate strand displacement activity. It was therefore proposed that both POL1A and POL1B can play a role in short-patch BER, while POL1B can also be implicated in long-patch BER.

Long-patch BER involves the displacement of several nucleotides and the processing of the 5′-flap (Figure 1). Up to now no plant mitochondrial 5′-flap endonuclease has been characterized, but the At3g52050 gene of Arabidopsis encodes a putative mitochondrial nuclease related to the 5′-3′ exonuclease domain of *E. coli* DNA polymerase I, which could potentially play this role.

A ligase is needed to finalize the process, which in Arabidopsis would be ligase 1, required for both short- and long-patch BER [124]. This ligase can be recovered in a short form in the nucleus or in a long form in mitochondria, due to the alternative use of two in-frame AUG start codons [125].

### 3.5. Recombination Pathways in Plant Mitochondria

Recombination is an important process shared by a wide range of organisms, both prokaryotic and eukaryotic, and even viral [126,127,128]. It allows the repair of DNA double-strand breaks (DSBs) and of single-strand gaps (SSGs), and is often required for correct progression of DNA replication because recombination activities are involved in the rescuing of stalled or damaged replication forks [129].

**Figure 2:**
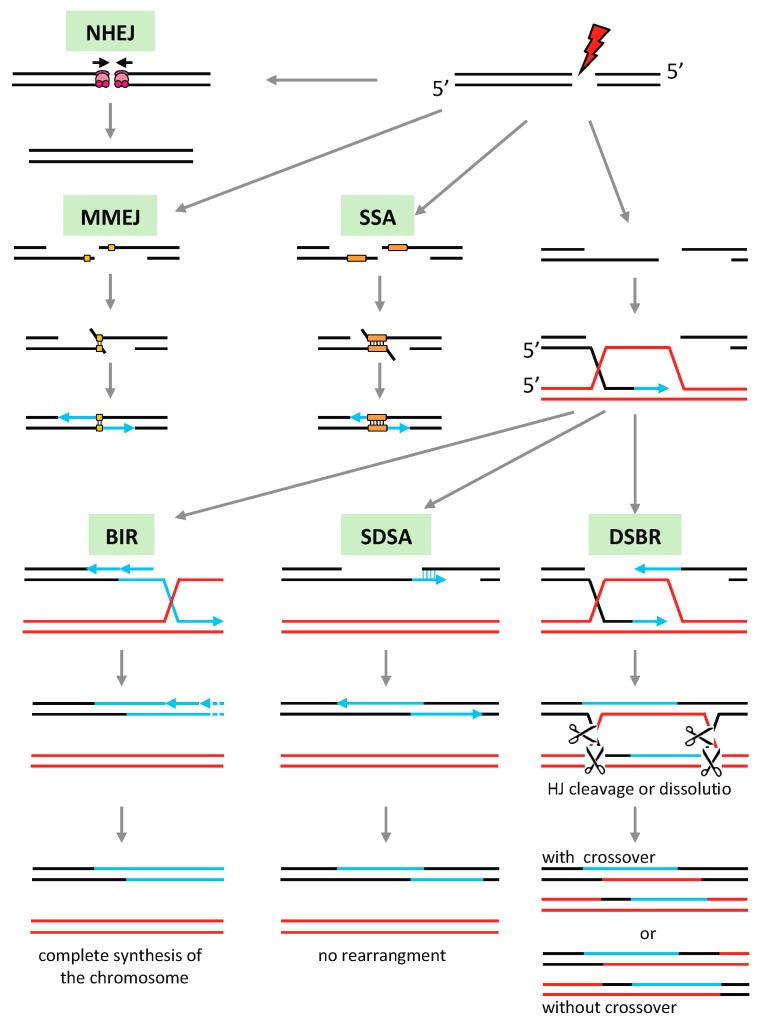
Repair of double-strand breaks (DSBs). The ends of a DSB can be repaired by non-faithful mechanisms independent of a recombinase. In the nucleus, non-homologous end-joining (NHEJ) involves Ku70, Ku80, and DNA-dependent protein kinase that bring together the DSB ends before they are religated. NHEJ generally leads to the suppression of several nucleotides on either side of the break. Microhomology-mediated end-joining (MMEJ) uses small homologous sequences (5–30 bp) to join both ends of a DSB and allow synthesis of missing complementary strands. By other pathways the DNA extremities are resected, giving protruding 3′ ends. If they contain complementary sequences they can base pair, leading to the deletion of the region between the repeats by single-strand annealing (SSA). Homology recombination (HR) pathways involve the invasion of homologous DNA by a nucleofilament of a recombinase (RecA/RAD51) bound to single-strand DNA (ssDNA). Several alternative pathways can be then followed. Double-strand break repair (DSBR) involves the recruitment of the second strand of the DSB and the formation of two Holliday junctions (HJ). The HJs can be resolved by dissolution, by branch migration towards each other and decatenation by a topoisomerase, or cleaved by a resolvase, leading to crossovers or no crossovers depending how they are resolved. By synthesis-dependent strand annealing (SDSA) the second end of the DSB is recruited after synthesis of the missing sequence using homologous DNA as template, leading to faithful repair without crossovers. The break-induced replication (BIR) pathway uses homologous DNA to re-synthesize DNA that has undergone DSB. BIR can lead to major genomic rearrangements when the homologous sequences are not allelic, such as with repeated sequences.

Among recombination mechanisms we have to distinguish between homologous and non-homologous pathways (Figure 2). Homologous recombination (HR) pathways rely on the identification of sequences with high similarity to the region to be repaired, typically other genome copies or large repeated sequences. They are the predominant mechanisms of repair in prokaryotes, also required for conjugation processes [130]. HR pathways are also found in the nucleus of eukaryotes, where they are regulated by the cell cycle that limits HR to the G2 and S phases when sister chromatids are available. This restriction promotes recombination between truly homologous sequences [131]. The different steps of HR are usually the same, although the factors involved may vary from organism to organism. The first step is the formation of a presynaptic nucleofilament between single-strand DNA (ssDNA) and a recombinase, RecA in bacteria and Rad51 in eukaryotes, capable of invading homologous double-strand DNA (dsDNA). This invasion, called synapse, is the second step. Finally, the post-synaptic steps involve the processing of the synapse and the separation of the two homologous DNA molecules.

Non-homologous recombination pathways such as non-homologous end-joining (NHEJ) or microhomology-mediated end-joining (MMEJ) utilize very limited or non-sequence similarities for the repair of broken DNA (Figure 2). They are the preferred mechanisms for the repair of DSB in somatic tissues of eukaryotes, although they may be deleterious to the genome since they can result in complex genome rearrangements, sequence deletions, and duplications [132,133]. Single-strand annealing (SSA) also involves base-pairing between sequences sharing significant homology, but in contrast to other HR pathways it does not require the intervention of a recombinase.

**Figure 3:**
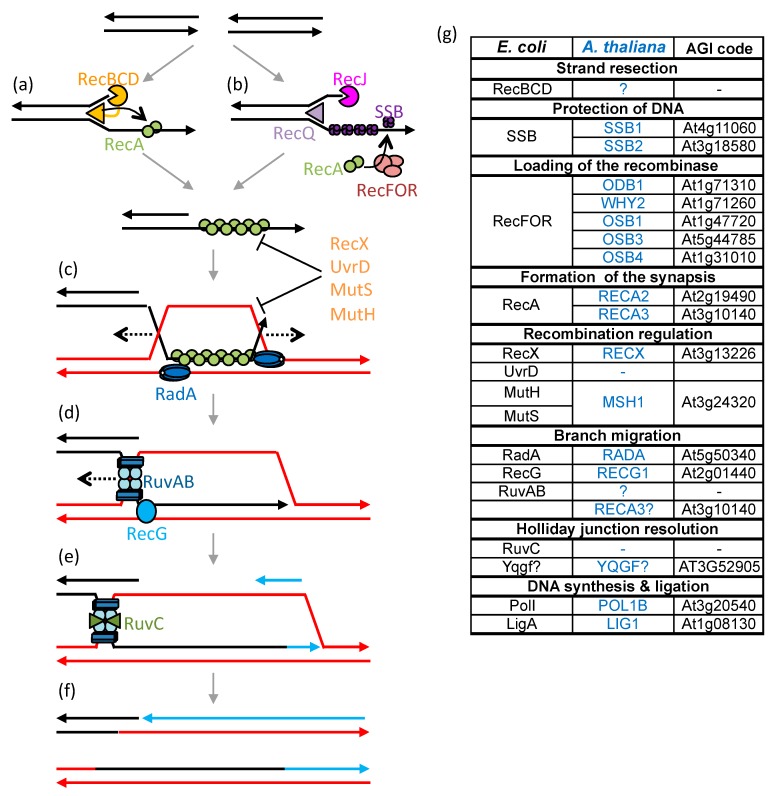
Different steps of bacterial homologous recombination (HR) and corresponding plant mitochondrial factors. The repair of double-strand breaks (DSBs) by HR begins with the resection of its ends. (**a**) The RecBCD pathway involves the RecBCD complex composed of two helicases and an exonuclease, and their actions lead to a single-strand DNA (ssDNA) loop region to which the RecA recombinases binds. (**b**) The alternative RecFOR pathway involves the RecQ helicase and the RecJ exonuclease that open and degrade the DNA, forming a protruding 3′ ssDNA end that is coated with the ssDNA-binding protein SSB. The RecFOR complex then promotes the replacement of SSB by RecA. After nucleation of RecA, the RecA-ssDNA nucleofilament is rapidly elongated and then scan for sequence homology in the genome. (**c**) When a homologous double-strand DNA (dsDNA) is found, the nucleofilament invades it and forms a three-stranded DNA structure called a D-loop. (**c**,**d**) Branch migration, early via RadA and later via translocases RecG and RuvAB, allows the extension of the homology region and the recruitment of the fourth strand, thus forming a Holliday junction (HJ). (**e**) The HJ is resolved, with or without a crossover, by the RuvC nuclease that is recruited by RuvAB. (**f**) A DNA polymerase synthetizes the missing DNA. (**g**) *Escherichia coli* factors and the probable functional homologues of *Arabidopsis thaliana* mitochondria.

In plant mitochondria HR is apparently the primary DNA repair pathway, while end-joining is rarely observed [134]. HR is also responsible for the rapid evolution of the genome organization, and has important roles in the replication and segregation of the mtDNA. Due to the high number of repeated sequences in the mtDNA, HR must be finely regulated to avoid genomic rearrangements that may be deleterious to the mitochondria. The mutation of factors involved in mitochondrial HR or its regulation usually leads to significant rearrangements of the plant mtDNA, by increased ectopic recombination involving repeats of intermediate size and microhomologies [63,78,134,135,136,137]. This could be because HR is important for replication-coupled repair, and if compromised there can be increased DNA breaks resulting from the collapse of replication forks. Replication would then be re-established following break-induced replication (BIR) (Figure 2) or by an MMEJ variant called microhomology-mediated BIR (MMBIR), both processes leading to gross genome rearrangements and instability [5]. Because the HR pathways of plant organelles are mainly derived from the prokaryotic ones, their study requires a good comprehension of the different steps of bacterial HR, as described in Figure 3.

In *E. coli*, the repair of double-strand breaks by HR begins with the resection of the 5′-phosphate ends of the break by the RecBCD helicase-exonuclease complex (Figure 3a). RecA then associates with the resulting single-stranded DNA (ssDNA) thus forming the presynaptic complex [138,139]. The DSB ends can also be processed by the 3′-5′ RecQ helicase and the RecJ 5′-3′ nuclease [140], which leads to 3′ ssDNA ends that are rapidly coated with SSB proteins (Figure 3b). SSB protects the ssDNA from degradation and removes secondary structures [141], but also inhibits RecA nucleation and therefore must be displaced to allow HR. The RecFOR complex mediates recombination, displacing SSB and allowing RecA loading on ssDNA [142,143]. RecFOR is also involved in the loading of RecA at SSGs resulting from replication defects. The HR repair of SSGs circumvents the stalling of the DNA polymerase and restores the progression of blocked replication forks [144]. The presynaptic complex scans dsDNA for a homologous sequence. For this the RecA nucleofilament associates randomly with multiple regions of the dsDNA till it meets a homologous sequence identified by Watson-Crick base pairing [145,146]. Invasion of the heterologous dsDNA by the RecA nucleofilament forms a triple-stranded structure called a D-loop (Figure 3c). The formation or maintenance of the D-loop may be inhibited by HR-regulating proteins, such as UvrD or RecX, depolymerizing or blocking the extension of the RecA nucleofilament, respectively [147,148].

The heteroduplex region is extended during the branch migration steps (Figure 3c,d), which ensure recombination between truly homologous sequences, whereas if the homology is weak the HR process is aborted [149]. Branch migration recruits several factors involved in partially redundant maturation pathways [150]. The first branch migration pathway involves RadA/Sms, a paralog of the RecA protein [150,151]. RadA hexamers are recruited by RecA and loaded on either side of the D-loop on a strand of the recipient dsDNA [151]. These hexamers have ATP-dependent helicase activity that unbinds the receptor dsDNA, extending the D-loop in both directions over long distances. The opening of the dsDNA receptor allows its pairing with the invading donor ssDNA. The second branch migration pathway involves the RecG helicase [152]. RecG is recruited by SSB proteins and acts as a monomer by unwinding the dsDNA receptor to hybridize it to the homologous complementary strand. Unlike RadA, RecG is an ATP-dependent helicase, which allows the recruitment of the second strand of invading DNA, converting the D-loop into a four-stranded DNA structure called the Holliday junction (HJ) [149]. Finally, HJs are processed by the RuvAB complex [153]. A RuvA tetramer binds specifically to the crossing of the four DNA strands, stabilizing its structure, and recruits RuvB on both sides of the HJ, which moves the HJ in one direction or the other, until recruitment of endonuclease RuvC that cleaves the HJ. The cleavage of the HJ allows the resolution of the heteroduplex via the separation of the two DNA molecules.

In plant organelles many of the factors involved in HR have been identified (Figure 3g), but several of the HR steps are still poorly understood. The early and late steps of the recombination process are particularly ill-defined because the proteins involved in the resection of DSB extremities and in the HJ resolution have not yet been identified. Also, many of the identified factors seem to have redundant activities, such as numerous ssDNA-binding proteins and several homologues of bacterial RecA, and it is difficult to distinguish their exact individual roles. These proteins might also function in alternative HR pathways, using different substrates and/or leading them to different outcomes.

Like in bacterial HR, after resection of DNA extremities ssDNA ends should be protected from nucleases by ssDNA-binding proteins. In plant mitochondria this function could be fulfilled SSB1 and/or SSB2, homologues of bacterial SSB. The possible differential functions of SSB1 and SSB2 are not known, but both are necessary for the recombination process [154]. The protection of ssDNA and the promotion/inhibition of HR could also be provided by organellar single-stranded binding (OSB) proteins or WHY proteins from the whirly family [134,135]. These proteins are directed to the mitochondria (OSB1, OSB4, WHY2), chloroplast (OSB2, WHY1, WHY3), or both organelles (OSB3), and have a higher affinity to ssDNA than SSB1 and SSB2 proteins [155]. The mutation of OSB1 and OSB4 leads to an increase in ectopic recombination involving repeats of intermediate size and to genomic rearrangements [135]. The loss of WHY also leads to an increase in recombination involving micro-homologies in the mitochondria (*why2* mutants) and in the chloroplast (double mutant *why1 why3*) and inhibits HR repair of ciprofloxacin-induced DSBs [134,156]. However, the OSB and WHY proteins cannot interact with the POL1B organellar polymerase, which is involved in repair mechanisms [155]. Garcìa-Medel et al. have therefore proposed alternative roles for SSB1/SSB2 and OSB/WHY proteins [155]. In DSB, the SSB1 and SSB2 proteins could promote the association of POL1B with the ends of the break, favoring repair by MMEJ, whereas OSB1 and WHY2 inhibit end-joining mechanisms by blocking the access of ssDNA 3′-OH ends to SSBs and DNA polymerases.

Loading the recombinase on the ssDNA to form the presynaptic filament requires replacement of the ssDNA binding proteins. The organellar DNA-binding (ODB) proteins are homologues of yeast Rad52 and could perform this function in the mitochondria and chloroplast, respectively [55,157]. As with Rad52, ODB1 promotes hybridization of complementary ssDNA sequences in vitro, supporting the mediator role of ODB1 in recombination [55]. In mitochondria, the WHY2 protein could also play the role of a mediator by promoting the loading of recombinases on ssDNA [134]. ODB1 and WHY2 could then be redundant or allow the recruitment of different factors involved in different alternative recombination pathways. ODB and WHY proteins are co-purified with organellar DNA and are considered as important components of nucleoids [41,158]. As for WHY2, loss of ODB1 inhibits HR repair of genotoxic-induced breaks, resulting in increased recombination involving micro-homologies [55,134]. In Arabidopsis, three recombinases homologous to bacterial RecA are addressed to organelles: RECA1 to the chloroplast, RECA3 to mitochondria and RECA2 to both organelles [92]. Interestingly, the orthologs of RECA3 are only found in angiosperms. In mitochondria, RECA2 and RECA3 appear to be involved in different HR pathways that may be partially redundant [137]. Thus, both RECA2 and RECA3 could partially complement the bacterial *recA-* mutant for survival after hydroxyurea and mitomycin C treatments, but only RECA2 could complement repair of damages induced by UV light. Both *recA2* and *recA3* mutants show an increase of ectopic recombination involving repeated sequences, but the *recA2* mutation is lethal early in seedling development, whereas *recA3* has little effect on the development of the plant in the first generation of homozygous mutants, but increases the sensitivity of the plant to genotoxic stress [137]. This suggests that RECA2 may be the main mitochondrial recombinases, whereas RECA3 rather participates in mtDNA repair, particularly in pathways that result in non-crossovers, such as synthesis-dependent strand annealing (SDSA) [137]. The absence of RECA2 would lead to excessive rearrangements of mtDNA due to increased ectopic recombination. In support of RECA2 and RECA3 being involved in different partially redundant pathways, the *recA3* mutation is highly synergistic with the mutation of other factors that modulate mitochondrial HR, such as MSH1 and RECG1 [78,136,159]. The Arabidopsis double mutant *odb1 recA3* is also very affected in their development (unpublished results of the laboratory).

RECA3 does not possess the important C-terminal acid extension found in all other RecA proteins, which in *E. coli* is necessary for the interaction between RecA and other factors that modulate its activity such as SSB and RecX [160,161]. RECA3 could thus escape regulation by mitochondrial factors that are active on RECA2. One of these could be RECX, the mitochondrial ortholog of bacterial RecX, which is found in all land plants. In agreement with this assumption it was recently shown that RECX from *Physcomitrella patens* interacts with mitochondrial RecA, and as expected for a RecA suppressor its overexpression mimics the RecA mutation [162]. Since there is no RECA3 in *Physcomitrella* it would be interesting to know if Arabidopsis RECX interacts with RECA2 or with RECA3, or with both.

As shown in Figure 3d, branch migration allows D-loops to be extended through the action of helicases. The RuvAB bacterial branch migration pathway is absent from plant organelles, since no orthologs of RuvA and RuvB are encoded by the genome of plants. In contrast, orthologs of the RecG and RadA proteins, described as partially redundant with RuvAB, are present in the genome of all plants. The RECG1 protein, homologue of RecG, is addressed to chloroplasts and mitochondria [63,163]. It partially complements the *recG* bacterial mutant in survival to UV treatment, suppression of pathological re-initiation of replication, and inhibition of replication using an RNA primer, implying that RECG1 performs the same functions in plant organelles as RecG in bacteria [63]. However, the *recG1* mutation has insignificant impact on plant development [63,163]. In bacteria, survival after genotoxic treatment is little affected when a single migration pathway is mutated, but survival decreases drastically when two pathways are affected [164]. With the absence of RuvAB in organelles, the absence of a developmental phenotype for *recG1* is rather surprising, especially with regard to the *recA2* mutation that is lethal at the seedling stage [137]. This suggests that another branch migration route plays a more important role in organellar HR. This pathway could involve RADA, the plant ortholog of eubacterial RadA. In agreement, we found that in Arabidopsis the RADA protein is addressed to mitochondria and chloroplasts, and that the loss of the *RADA* gene has severe effects on mtDNA stability and plant development [165]. It is also possible that an additional factor evolved to assume branch-migration functions, and RECA3 could be such a factor. Indeed, one of the intrinsic activities of RecA proteins is branch migration, which allows strand exchange reactions to take place in vitro, in the presence of RecA only. The deletion from *E. coli* recA of the acidic 17 aa C-terminal sequence virtually affects every RecA function, with almost all activities being more robust when it is absent [166]. Among others, the deletion mutant no longer requires free Mg^2+^ ion for optimal strand exchange activity, and binds faster to duplex DNA [167,168]. It is therefore possible that RECA3, lacking the C-terminal acidic extension, might have increased branch-migration activity and be able to partially complement the absence of RECG1 or RADA in plant mitochondria.

The absence of a RuvAB pathway in organelles also results in the absence of the RuvC resolvase involved in HJ resolution. However, not all bacteria use RuvC, but alternative resolvases such as RecU or YqgF [127,169]. A homologue of YqgF is found in plants and could potentially be involved in the resolution of HJ in organelles. In contrast, in the chloroplasts, a nuclease able to resolve HJs has been identified [170]. This resolvase, monokaryotic chloroplast 1 (MOC1), is only targeted to chloroplasts and there is no other isoform of MOC1 coded by plant genomes that could be the resolvase in charge of mitochondrial HJs. Mutation of *MOC1* results in a loss of the cellular cpDNA and in aberrant chloroplast nucleoid segregation, suggestive of the importance of HJ resolution for the proper segregation of the organellar genomes. Alternatively, it is also possible that mitochondrial HJs are not resolved, but undergo dissolution due to the combined actions of branch migration factors and topoisomerase I, as in nuclear HR, or that RECG1 can resolve HJs by moving them to replication forks, as proposed for bacterial RecG [171].

## 4. HR Pathways and the Evolution of the Plant mtDNA

The mitochondrial genomes of plants generally have a very low mutation rate compared with the nuclear or mitochondrial genomes of animals and yeasts [18]. On the other hand, they show an excessively high rate of genomic rearrangement, leading to poor synteny conservation, even within the same species [17]. The structural evolution of the mtDNA is very rapid and can occur from one generation to the next, and can lead to particular phenotypic modifications such as cytoplasmic male sterility (CMS) [172,173].HR is the process responsible for this evolution, as well by correcting genetic mutations faithfully as by promoting genomic rearrangements [77,174].

If the mtDNA gene sequences are highly conserved, the non-coding sequences are much less so. These regions are difficult to align because they vary widely from one species to another, which complicates the study of their mutation rates. Nevertheless, by comparing the mtDNA of two ecotypes of Arabidopsis that diverged recently it has been shown that non-coding sequences have a higher mutation rate than coding sequences [77]. However, mechanisms for the preferential repair of transcribed regions have not been evidenced in plant organelles. This conservation of coding regions would rather result from the specific selection of mtDNA molecules that do not exhibit functional alterations [77]. The selection pressure on non-coding regions being virtually absent, their mutation would not be counter-selected. It has also been proposed that mtDNA damage is preferentially repaired by HR [77]. Recombination involving large repeats is frequent and reversible, and is responsible for the normal multipartite organization of the plant mtDNA. However, under stress, smaller repeats may be mobilized by less faithful repair mechanisms such as BIR and MMEJ [55,134,137]. These mechanisms will induce deletions or, more frequently, duplications of sequences [77]. Unless these rearrangements are deleterious to mitochondrial function they will be retained, leading to a general increase in the mtDNA size [77].

In animals, some of the mitochondria from female oocytes, but not from male gametes, are small and suppressed for transcription, electron transport, and free radical production. This would be a mechanism to prevent DNA damage and increasing the fidelity of mitochondrial DNA inheritance [175]. Plants, however, would have retained a mechanism to faithfully correct gene sequences, in exchange for increasing the mtDNA size [77]. This mechanism could be at the origin of the wide range of mtDNA sizes in the *Silene* family, ranging from 253 kb for *Silene latifolia,* to the largest known mtDNA of 11.3 Mb for *Silene conica* [15]. These large genomes also have an exceptionally high mutation rate, even surpassing that of animal mtDNA, while the smaller *Silene* genomes have a low mutation rate, as in most flowering plants [15,25]. The large genomes consist of a multitude of independent chromosomes, up to more than 50, which may contain no identifiable genes. Since there is no functional selection to retain them, these subgenomes can be rapidly acquired or lost in the same species [176].

In the absence of stress, ectopic recombination mediated by intermediate-size repeats is infrequent and appears to depend on repeat length as well as on their position in the genome [78,137]. The smaller the repeat size, less frequent and irreversible is recombination [177], but not strictly. The propensity for recombination involving repeats also depends on how far they are on the genome and if they are in two different subgenomic molecules (intermolecular recombination) or in a single DNA molecule (intramolecular recombination). Alternative mtDNA versions generated through short homologies are still detected in subliminal amounts, usually less than one per cell [54,56]. These sublimons contribute to the heteroplasmy of the mitochondrial genome. Nevertheless, these low-copy versions may become the predominant mtDNA, in a process of clonal expansion called sub-stoichiometric shift (SSS) [56,178,179]. SSS happens rapidly, from one generation to the next, and is responsible for the rapid evolution of the plant’s mtDNA structure. The mechanisms responsible for SSS are not completely clear. SSS results from the preferential replication or segregation of specific subgenomes, a process that apparently also depends on HR pathways, as suggested by the segregation of subgenomes resulting from recombination in the backcross of *recG1* mutants [63]. Mutants of factors involved in HR and its regulation show a higher incidence of SSS, apparently related to increased ectopic recombination [63,135]. The fusion of mitochondria harboring different genomes, like in cybrids, also elicits recombination and preferential segregation of one type of rearranged mtDNA [180,181,182]. Like SSS in recombination mutants, the main recombination mechanism that was most frequent recognized in cybrid mitochondria was the BIR pathway, recruiting both large and intermediate size repeats [181,182].

Genomic rearrangements resulting from ectopic recombination may be of no consequence for mitochondria when they only affect non-coding regions and sequences not involved in gene regulation. However, they can also disrupt coding sequences, create chimeric genes, or displace regulatory regions allowing the expression of non-transcribed sequences [183]. These rearrangements can cause developmental problems or lethality, but can also bring new beneficial functions [184]. In particular, expression of chimeric genes can be responsible for CMS lines unable to produce viable pollen, which are sought by breeders for the production of hybrid seeds [172,185]. These chimeric genes allow the expression of a protein deleterious for mitochondrial function, called sterilizing factor, directly affecting the formation of anthers or the production of pollen, which are energy-intensive processes for the cell [172]. In natural populations CMS leads to gynodioecy, the co-occurrence of female and hermaphroditic individuals within a population [186]. The reason for the maintenance of the CMS phenotype would be the competitive advantage of sparing the energy spent in the formation of the male reproductive system [172].

In the chloroplast the genome is significantly less subject to the type of rearrangements observed in the mtDNA, because apart from the large inverted repeat found in the cpDNA of most plants there are few other repeated sequences in the cpDNA. DSBs in the cpDNA are repaired by intermolecular recombination (gene conversion) leading to the conservation of chloroplast sequences [187]. However, MMEJ and to a lesser extent NHEJ may also participate in the repair of DSBs, leading to the loss or duplication of large genomic regions [188,189]. MMEJ was also found as responsible for cpDNA rearrangements in *why1 why3* mutants [156,190]. Mutation of the cpDNA can also result from the sliding of DNA polymerases on short tandem-repeated sequences during replication [191]. However, in mosses such as *Physcomitrella* or in algae such as *Chlamydomonas* there is a greater incidence of short repeat sequences in the cpDNA, making it more susceptible to rearrangements [192,193].
